# Synergistic Effect of Combination Treatment of Brexpiprazole and Nalmefene on Ethanol Intake in Rats

**DOI:** 10.1002/npr2.70107

**Published:** 2026-03-06

**Authors:** Naoki Amada, Mai Nakamura, Yuta Ohgi, Yusuke Kakumoto, Mikio Suzuki, Takashi Futamura, Kenji Maeda

**Affiliations:** ^1^ Department of CNS Research, Tokushima Research Center for Drug Discovery Otsuka Pharmaceutical Co. Ltd. Tokushima Japan; ^2^ Department of Neurodegenerative Disease Research, Osaka Research Center for Drug Discovery Otsuka Pharmaceutical Co. Ltd. Osaka Japan; ^3^ Department of Lead Discovery Research Otsuka Pharmaceutical Co. Ltd. Tokushima Japan; ^4^ CNS Group, Medical Affairs Department Otsuka Pharmaceutical Co. Ltd. Tokyo Japan

**Keywords:** alcohol dependence, alcohol use disorder, combination treatment, limited access paradigm

## Abstract

**Aims:**

Reduction of alcohol consumption is one of treatment goals to reduce harm among individuals with alcohol use disorder (AUD), a major worldwide health problem, for which nalmefene, an opioid receptor modulator, is used. In this study, the effect of nalmefene on ethanol (EtOH) intake, already reported, was evaluated in Wistar rats, as validation. In addition, effects of brexpiprazole, serotonin–dopamine activity modulator, alone and in combination with nalmefene were evaluated to investigate further treatment option for AUD.

**Methods:**

During the first training phase, animals had 10% EtOH as the only drinking fluid available for the first 5 days. Then, the animals had a 24‐h free choice between EtOH and water for 39 days which is named the continual access paradigm. Thereafter, the limited access paradigm, which restricted the availability of 10% EtOH to 1 h every day, was carried out for 114 days. EtOH intake (g/kg/1 h) was determined by weighing 10% EtOH bottles before and after the limited EtOH access every day. Brexpiprazole (0.01–0.1 mg/kg, orally) and nalmefene (0.04–0.4 mg/kg, subcutaneously) were daily administered to rats 1 h or 20 min before starting the limited access paradigm for consecutive 4 days, respectively. The combination effect was evaluated using each subeffective dose of brexpiprazole and nalmefene which did not significantly reduce EtOH intake. The daily and the average EtOH intake for 4 days before and during the treatment with test compounds were statistically analyzed.

**Results:**

Brexpiprazole (0.1 mg/kg) and nalmefene (0.4 mg/kg) alone significantly decreased EtOH intake. Moreover, the combination of subeffective doses of brexpiprazole (0.01 mg/kg) and nalmefene (0.04 mg/kg) significantly and synergistically decreased EtOH intake.

**Conclusion:**

These data suggest that brexpiprazole may have the potential to decrease alcohol intake in AUD patients. In addition, brexpiprazole may have a synergistic therapeutic effect with nalmefene in those patients.

## Abstract 2

Combination effect of nalmefene and brexpiprazole on ethanol intake in rats. The combination of subeffective doses of nalmefene (0.04 mg/kg, s.c.) and brexpiprazole (0.01 mg/kg, p.o.) significantly and synergistically decreased the average ethanol intake for 4 days compared with before the treatment (interaction effect: *p* = 0.0033 by two‐way ANOVA).

## Graphical abstract

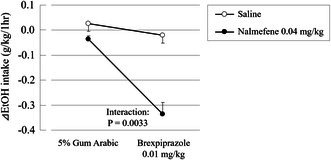

## 1 Introduction

Alcohol use disorder (AUD), characterized by a large consumption of alcoholic beverages, is known as a global major health problem [1, 2]. The WHO estimates 400 million people, or 7% of the world population ≥ 15 years old, have AUD [1], of which 209 million people (3.7% of the adult world population) live with alcohol dependence. Approximately 3 million deaths are annually caused by alcohol consumption in the world [1, 2], of which 2 million deaths were in men and 0.6 million deaths in women in 2019. Alcohol is widely used over the world and associated with significant health risks such as cardiovascular diseases, cancers, liver diseases, heart diseases, mental illnesses, and injuries [1, 3].

For the treatment of AUD, disulfiram, an alcohol deterrent, and acamprosate, a drug to maintain alcohol abstinence, are approved by the US Food and Drug Administration (FDA) and the European Medicines Agency (EMA) [3]. Similarly, nalmefene is approved by the EMA for the reduction of alcohol consumption in adult patients with alcohol dependence who have a high drinking risk level, without physical withdrawal symptoms and who do not require immediate detoxification [4, 5]. Nalmefene is also approved in Japan in 2019 [6]. From the perspective of harm reduction, nalmefene is used for reduction of alcohol consumption in alcohol‐dependent patients. Nalmefene is an opioid system modulator with antagonist activity at the μ and the δ receptors and partial agonist activity at the κ receptor [7]. The efficacy of as‐needed use of nalmefene has been demonstrated in two 24 week‐ and one 52 week‐randomized, double‐blind, placebo‐controlled, parallel‐group multicenter Phase 3 trials in patients with alcohol dependence, in which nalmefene showed a significant decrease of heavy‐drinking days and/or total alcohol consumption [4, 7, 8].

Comorbidity of AUD in psychiatric disorders is known with lifetime prevalence rates between 20% and 30%, especially in schizophrenia with a threefold increased risk compared to the general population [9]. This is also associated with an increase in the frequency and length of hospitalization of patients with schizophrenia. In addition to schizophrenia, comorbidity of alcohol dependence with anxiety disorder, depressive disorder, and post‐traumatic stress disorder (PTSD) has also been known [10, 11, 12].

Brexpiprazole is a serotonin–dopamine activity modulator which is a partial agonist at serotonin 5‐HT_1A_ and dopamine D_2_ receptors at relatively equal potency and an antagonist at 5‐HT_2A_ and noradrenaline alpha_1B_ and alpha_2C_ receptors [13]. While brexpiprazole has not been approved for the treatment of AUD, it was approved as treatment for schizophrenia and as an adjunctive therapy for major depressive disorder by the FDA in 2015 [14]. The FDA also approved an additional indication of brexpiprazole for the treatment of agitation in Alzheimer's dementia in 2023 [15]. Brexpiprazole is also approved for these indications in Japan [16].

Sinclair reported that nalmefene at 0.36 mg/kg significantly suppressed ethanol (EtOH) intake in rats [17]. Reading the graph data in Sinclair's report, the average reduction of EtOH intake after 4‐day nalmefene treatment period from 4‐day pre‐treatment period was approximately 0.3–0.4 g/kg/1 h (approximately 60% reduction from the pre‐treatment period). In the present study, the effect of nalmefene on ethanol (EtOH) intake was evaluated in Wistar rats, using the limited access paradigm as a validation. In addition, we investigated the potential of brexpiprazole to suppress EtOH intake in Wistar rats to investigate further treatment options. First, the effect of brexpiprazole treatment alone was evaluated. Then, the combination effect of brexpiprazole and nalmefene on EtOH intake was evaluated using doses which each compound alone did not suppress EtOH intake.

## 2 Materials and Methods

### 2.1 Animals

Male Wistar rats (Japan SLC Inc., 24 rats, 9 weeks old at time of starting EtOH intake training, for measurement of EtOH intake; 16 rats, 15–18 weeks old, for measurement of spontaneous locomotor activity) were used. Rats were single‐housed in individual transparent plastic cages (M85, width 31.4 cm × height 20.1 cm × depth 46.8 cm, Edstrom Japan Co. Ltd.) for precise measurement of EtOH consumption. For measurement of spontaneous locomotor activity, rats were group‐housed (3–4 rats per cage) in transparent plastic cages.

All animals had free access to water and food (MF, Oriental Yeast Co Ltd., Tokyo, Japan) supplied ad libitum during the study, except for the situations noted below, and maintained under artificial lighting between 7:00 a.m. to 7:00 p.m. The room temperature and humidity were maintained at 23°C ± 2°C and 60% ± 10%, respectively.

### 2.2 Drugs

Ten percentage (v/v) EtOH solution was prepared by diluting 99.5% EtOH (Fujifilm WAKO pure chemical corporation, Osaka, Japan) with water. Nalmefene hydrochloride (Tocris Bioscience, Bristol, UK) was dissolved and diluted with saline. Brexpiprazole, synthesized at Otsuka Pharmaceutical Co. Ltd., was suspended in 5% (w/v) gum arabic‐distilled water solution and diluted with the same solution. Nalmefene and saline were subcutaneously (s.c.) injected to rats at a volume of 1 mL/kg body weight. Brexpiprazole and 5% gum arabic solution were orally (per os; p.o.) administered to rats at a volume of 5 mL/kg body weight. The doses of brexpiprazole and nalmefene were chosen based on previous reports [17, 18].

### 2.3 Limited Access EtOH‐Drinking Paradigm

**FIGURE 1:**
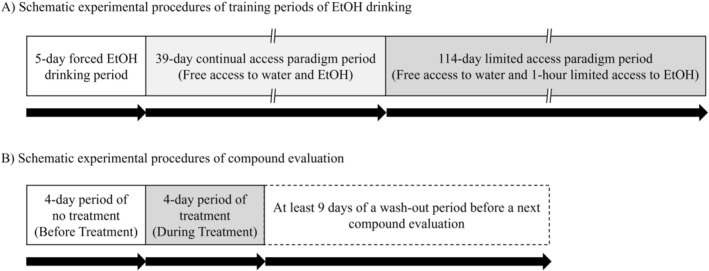
Schematic diagram of experimental procedures. (A) For training of EtOH drinking, rats underwent a 5‐day forced EtOH drinking period, a 39‐day continual access paradigm period, and a 114‐day limited access paradigm period. In the 5‐day forced EtOH drinking period, rats were given only 10% EtOH solution as available drinking fluid. In the 39‐day continual access paradigm period, the rats were given free access to water and 10% EtOH solution. In the 114‐day limited access paradigm period, the rats were given free access to water and a 1‐h limited access to 10% EtOH solution. (B) Within the 114‐day limited access paradigm period, for every compound evaluation, test rats underwent a 4‐day period of no treatment (before treatment), and a 4‐day period of treatment (during treatment) in which rats were treated with a test compound (nalmefene or brexpiprazole) or vehicle (saline or 5% gum arabic solution). Then, rats were given a washout period for at least 9 days before a next compound evaluation.

Animals were trained to voluntarily drink 10% EtOH solution with reference to the methods previously reported by Sinclair et al. [19]. First, 24 rats (body weight: 287.00–369.54 g) were divided into two groups based on body weight by a stratified random sampling to avoid allocation of animals with large differences between the groups: one is water group (*n* = 8) and the other was EtOH group (*n* = 16). The EtOH group underwent a 5‐day forced EtOH‐drinking period in which the only available drinking fluid was 10% EtOH solution (schematic experimental procedures are shown in Figure 1). Thereafter, one tap water bottle per cage (and food) was always available. Then, the continual access paradigm was carried out. In the continual access paradigm period, the animals had a 24‐h two bottle free choice between 10% EtOH solution and water until ethanol intake reached steady state (range of 0.60–1.06 g/kg/day), which took about 6 weeks. The amount of EtOH intake (g/kg/day) was determined by measuring the weight of bottles containing 10% EtOH solution every day and by calculating the EtOH amount from the weight. Positions of the bottles of 10% EtOH and water on the cages were exchanged every day. There was no difference in body weight between the EtOH group and the water group.

After the training period, the rats underwent the limited access paradigm in which rats were given free access to water and 1‐h limited access to 10% EtOH every day, between 8 a.m. and 4 p.m. during the daytime. The amount of EtOH intake (g/kg/1 h) was determined every day by measuring the weight of bottles containing 10% EtOH solution before and after the limited EtOH access, and by calculating the EtOH amount from the weight. In each test for evaluation of test compounds or vehicle (5% gum arabic or saline) treatment, 5 or 6 test animals were selected from 10% EtOH trained rats based on the average amount of EtOH intake for 4 days before testing in the limited access paradigm with a cutoff amount of 0.4 g/kg/1 h. The drug‐free washout periods (at least 9 days) between the tests were employed in order to repeatedly use animals by eliminating the effects of previously administered test compounds. EtOH intake and water intake were measured every day.

### 2.4 Evaluation of the Effects of Nalmefene and Brexpiprazole on EtOH Intake

Rats were weighed prior to each limited access drinking session in order to calculate the administration volume of test compounds.

In order to investigate effective and subeffective doses of nalmefene and brexpiprazole prior to conduction of combination treatment experiment, we evaluated three doses of each compound (nalmefene: 0.04, 0.12, and 0.4 mg/kg, brexpiprazole: 0.01, 0.03, and 0.1 mg/kg). For these individual dose evaluations, nalmefene and saline were daily s.c. administered 20 min before starting the limited access paradigm on consecutive 4 days. Brexpiprazole and 5% gum arabic solution were daily p.o. administered 1 h before starting the limited access paradigm on consecutive 4 days.

Combination treatment experiment of brexpiprazole with nalmefene was divided into two experiments and conducted with attention in avoiding assignment of animals into the same treatment group (*n* = 3 rats × 2 experiments = 6 rats/treatment condition). For combined treatment, rats were daily treated with nalmefene (s.c.) or saline (s.c.) 20 min before starting the limited access paradigm, and brexpiprazole (p.o.) or 5% gum arabic solution (p.o.) 60 min before starting the limited access paradigm on consecutive 4 days.

Daily EtOH intake was recorded for 4 days before the test compound treatment and 4 days during the test compound treatment. Then, the average EtOH intakes for the 4 days before the test compound treatment and the 4 days during the test compound treatment were calculated and used to statistically analyze the effect of the compound treatment.

### 2.5 Spontaneous Locomotor Activity

In order to measure the locomotor activity, SUPERMEX (Muromachi Kikai Co. Ltd.) was used with a plastic cage (M85) in a soundproof room. The locomotor activity was measured in animals different from those used for the EtOH intake evaluation. One day before testing, the body weight of each animal was measured for grouping. Based on the body weight, the administration volume of test compounds was calculated. Nalmefene or saline were s.c. administered 20 min before starting the locomotor activity measurement. Spontaneous locomotor activity was measured for 1 h. The total counts of locomotor activity of animals were used to assess the effect of nalmefene.

### 2.6 Statistical Analysis

All statistical analyses were conducted using SAS Software for Windows, Release 9.3 (SAS Institute Japan Ltd., Tokyo, Japan). Data are presented as mean ± SEM. All analyses were two‐sided, and *p* values < 0.05 were considered statistically significant.

In the investigation of effective and subeffective doses of each compound, the differences of the average EtOH intake over 4 days between before and during treatment with each test compound or the corresponding vehicle were analyzed using a paired *t*‐test. Differences in daily EtOH intakes over consecutive 4 days before and during treatment with each test compound or vehicle were analyzed using a mixed effect model for repeated measures (MMRM), with animal ID, time (day), and treatment period (before or during treatment) included as factors. Differences in daily EtOH intakes between vehicle treatment [saline (s.c.) or 5% gum arabic solution (p.o.)] and nalmefene (0.04, 0.12, or 0.4 mg/kg, s.c.) or brexpiprazole (0.01, 0.03, or 0.1 mg/kg, p.o.) treatment were analyzed using MMRM, with animal ID, time (day), and treatment condition included as factors, and baseline EtOH intake (the average intake over the 4 days before treatment) included as a covariate. Comparisons between vehicle and each treatment group were performed using Dunnett's multiple comparison procedure based on the fitted MMRM.

In the combination treatment study of brexpiprazole and nalmefene, differences in daily EtOH intake over consecutive 4 days before and during each combination treatment were analyzed using a MMRM, with animal ID, time (day), and treatment period (before or during treatment) included as factors. Differences between the brexpiprazole (0.01 mg/kg, p.o.) + nalmefene (0.04 mg/kg, s.c.) combination treatment and other treatment conditions [5% gum arabic (p.o.) + saline (s.c.), 5% gum arabic (p.o.) + nalmefene (0.04 mg/kg, s.c.), and brexpiprazole (0.01 mg/kg, p.o.) + saline (s.c.)] were analyzed using MMRM, with animal ID, time (day), and treatment condition included as factors, and baseline EtOH intake included as a covariate. Dunnett's multiple comparison procedure was applied with the brexpiprazole + nalmefene combination treatment specified as the control group. In addition, the delta values of the average EtOH intake for 4 days between before and during the treatment were calculated in each animal, and a two‐way analysis of variance (ANOVA) was performed to evaluate the interaction effect between brexpiprazole (presence or absence) and nalmefene (presence or absence).

The difference of the total counts of spontaneous locomotor activity between vehicle group (saline) and each dose group of nalmefene was assessed using Dunnett's test.

## 3 Results

### 3.1 Effect of Nalmefene and Brexpiprazole Alone on EtOH Intake in Limited Access Paradigm

In the evaluation of nalmefene's effects, percent reductions of the average EtOH intake for the 4 days during the treatment from 4 days before the treatment were as follows: saline (s.c.), −7.8%; nalmefene 0.04 mg/kg (s.c.), 3.4%; nalmefene 0.12 mg/kg (s.c.), 26.3%; and nalmefene 0.4 mg/kg (s.c.), 62.8%.

**FIGURE 2:**
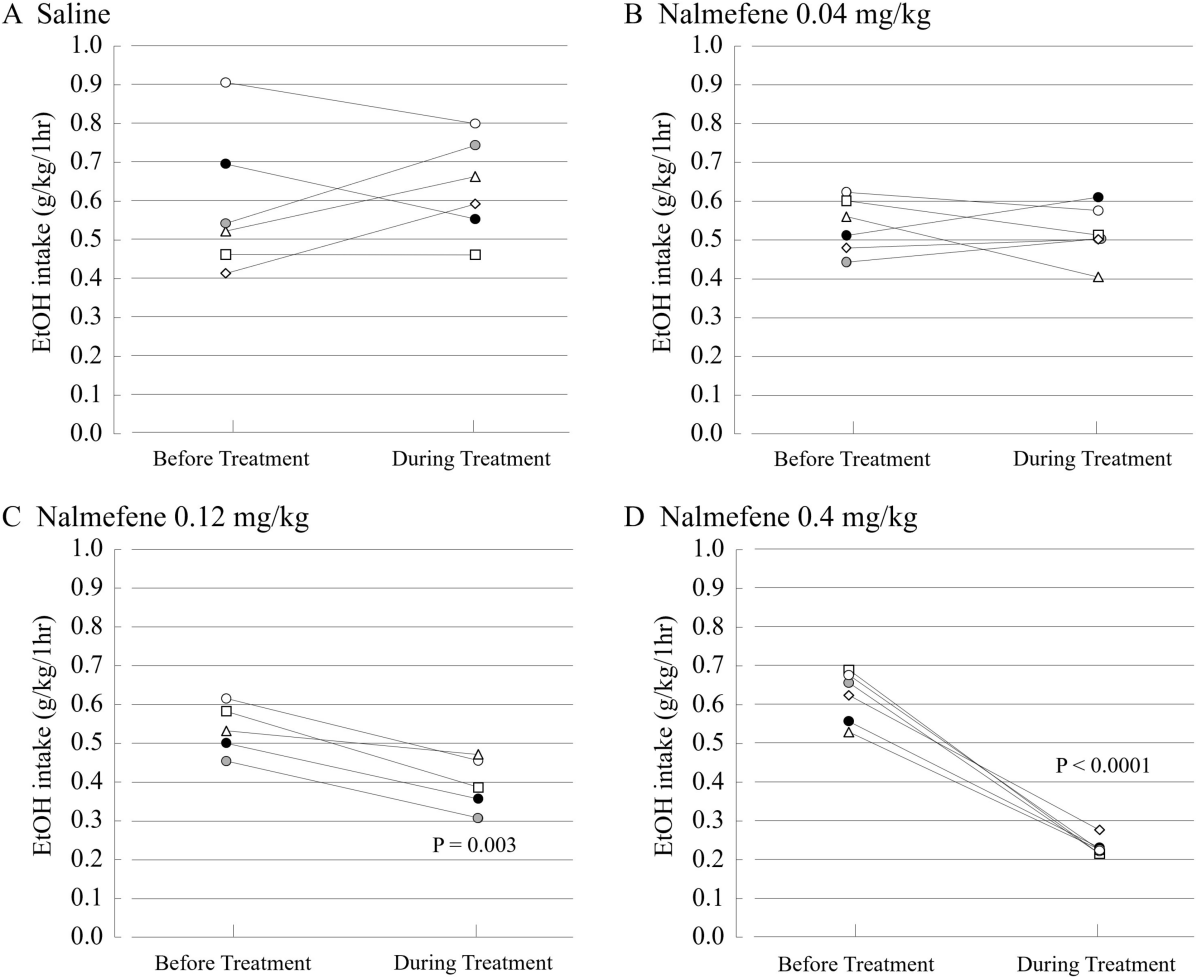
Effect of nalmefene on the average EtOH intake in limited access paradigm in rats. Average EtOH intake in rats treated with (A) saline (s.c.), (B) nalmefene 0.04 mg/kg (s.c.), (C) nalmefene 0.12 mg/kg (s.c.), and (D) nalmefene 0.4 mg/kg (s.c.). Data are presented as mean ± SEM (*n* = 5–6). The differences of the average EtOH intake for 4 days between before and during the treatment of nalmefene (0.04, 0.12, 0.4 mg/kg, s.c.) or saline were analyzed using a paired *t*‐test. Nalmefene at doses of 0.12 and 0.4 mg/kg (s.c.) indicated the statistically significant decreasing effect on EtOH intake in limited access paradigm in rats (*p* = 0.003 and *p* < 0.0001, respectively).

As shown in Figure 2, nalmefene significantly decreased the average EtOH intake in the limited access paradigm for 4 days during the treatment at doses of 0.12 and 0.4 mg/kg (s.c.) compared with the average EtOH intake for 4 days before the treatment (*p* = 0.003 and *p* < 0.0001, respectively). The lowest dose of nalmefene (0.04 mg/kg, s.c.) did not show statistically significant effects.

The effects of nalmefene on daily EtOH intakes over the treatment period of consecutive 4 days were also analyzed. Nalmefene at 0.12 and 0.4 mg/kg (s.c.) significantly decreased daily EtOH intakes over the 4‐day treatment period compared with the 4‐day period before the treatment (*p* = 0.0001 and *p* < 0.0001, respectively; Figure S1). Nalmefene at 0.04 mg/kg (s.c.) did not significantly decrease daily EtOH intakes.

**FIGURE 3:**
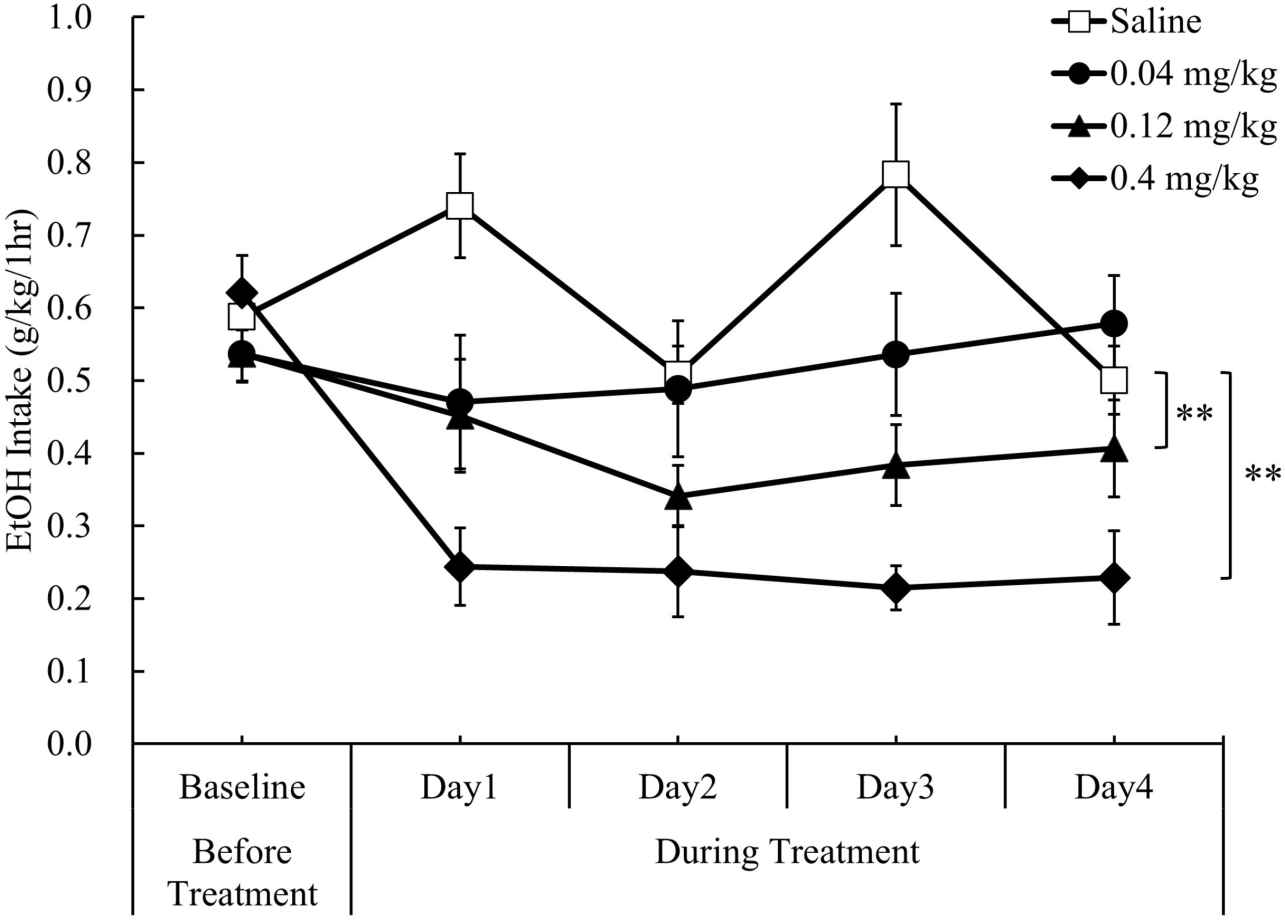
Effect of nalmefene on daily EtOH intake fluctuations in limited access paradigm in rats. Differences in daily EtOH intakes over the 4‐day treatment period between saline and nalmefene (0.04, 0.12, or 0.4 mg/kg, s.c.) were analyzed using a mixed effect model for repeated measures (MMRM), with animal ID, time (day), and treatment condition included as factors, and baseline EtOH intake, defined as the 4‐day average before treatment, included as a covariate. Comparisons with saline were performed using Dunnett's multiple comparison procedure. Nalmefene at doses of 0.12 and 0.4 mg/kg (s.c.) significantly reduced daily EtOH intakes compared with saline over the 4‐day treatment period in the limited access paradigm in rats. Data are presented as mean ± SEM (*n* = 5–6). **p < 0.01 vs. saline.

Moreover, there were significant differences in daily EtOH intake fluctuations for consecutive 4‐day treatment periods between saline treatment and nalmefene 0.12 mg/kg treatment (*p* = 0.0004; Figure 3), and between saline treatment and nalmefene 0.4 mg/kg treatment (*p* < 0.0001; Figure 3).

Based on these results, the dose at 0.04 mg/kg (s.c.) was defined as a subeffective dose of nalmefene on EtOH intake in the limited access paradigm for combination study with brexpiprazole.

In the evaluation of brexpiprazole's effects, percent reductions of the average EtOH intake for the 4 days during the treatment from 4 days before the treatment were as follows: 5% gum arabic (p.o.), −2.5%; brexpiprazole 0.01 mg/kg (p.o.), 14.5%; brexpiprazole 0.03 mg/kg (p.o.), 15.2%; and brexpiprazole 0.1 mg/kg (p.o.), 58.4%.

**FIGURE 4:**
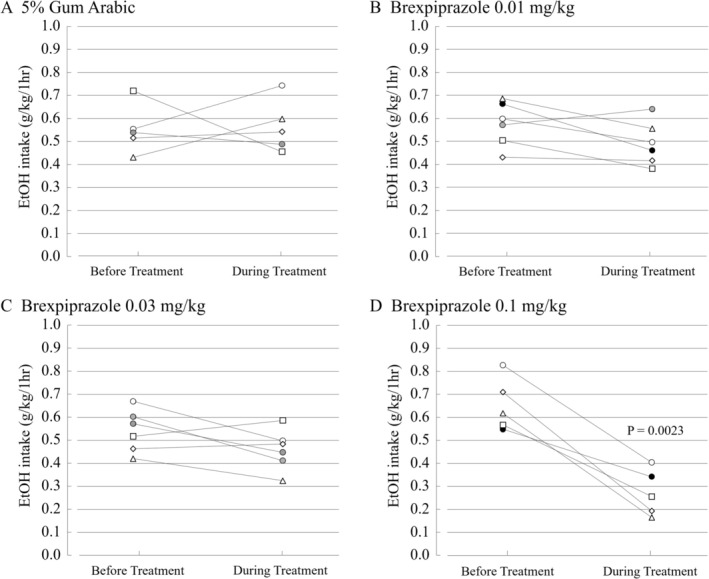
Effect of brexpiprazole on the average EtOH intake in limited access paradigm in rats. Average EtOH intake in rats treated with (A) 5% gum arabic (p.o.), (B) brexpiprazole 0.01 mg/kg (p.o.), (C) brexpiprazole 0.03 mg/kg (p.o.), and (D) brexpiprazole 0.1 mg/kg (p.o.). Data are presented as mean ± SEM (*n* = 5–6). The differences of the average EtOH intake for 4 days between before and during the treatment of brexpiprazole (0.01, 0.03, 0.1 mg/kg, p.o.) or 5% gum arabic were analyzed using a paired *t*‐test. Brexpiprazole at doses of 0.1 mg/kg (p.o.) indicated the statistically significant decreasing effect on EtOH intake in the limited access paradigm in rats (*p* = 0.0023).

As shown in Figure 4, brexpiprazole significantly decreased the average EtOH intake in the limited access paradigm for 4 days during the treatment at the dose of 0.1 mg/kg (p.o.) compared with the average EtOH intake for 4 days before the treatment (*p* = 0.0023). Low and medium doses of brexpiprazole (0.01 and 0.03 mg/kg, p.o.) did not show statistically significant effects. However, when the average EtOH intake for 4 days during treatment of brexpiprazole was compared with the average EtOH intake for 4 days after the treatment (data not shown), the average EtOH intake for 4 days after the treatment of brexpiprazole at 0.03 mg/kg (p.o.) significantly increased (*p* < 0.01 by the paired *t*‐test) compared with the average EtOH intake for 4 days during the treatment (data not shown), indicative of EtOH intake reduction during brexpiprazole 0.03 mg/kg (p.o.) treatment.

**FIGURE 5:**
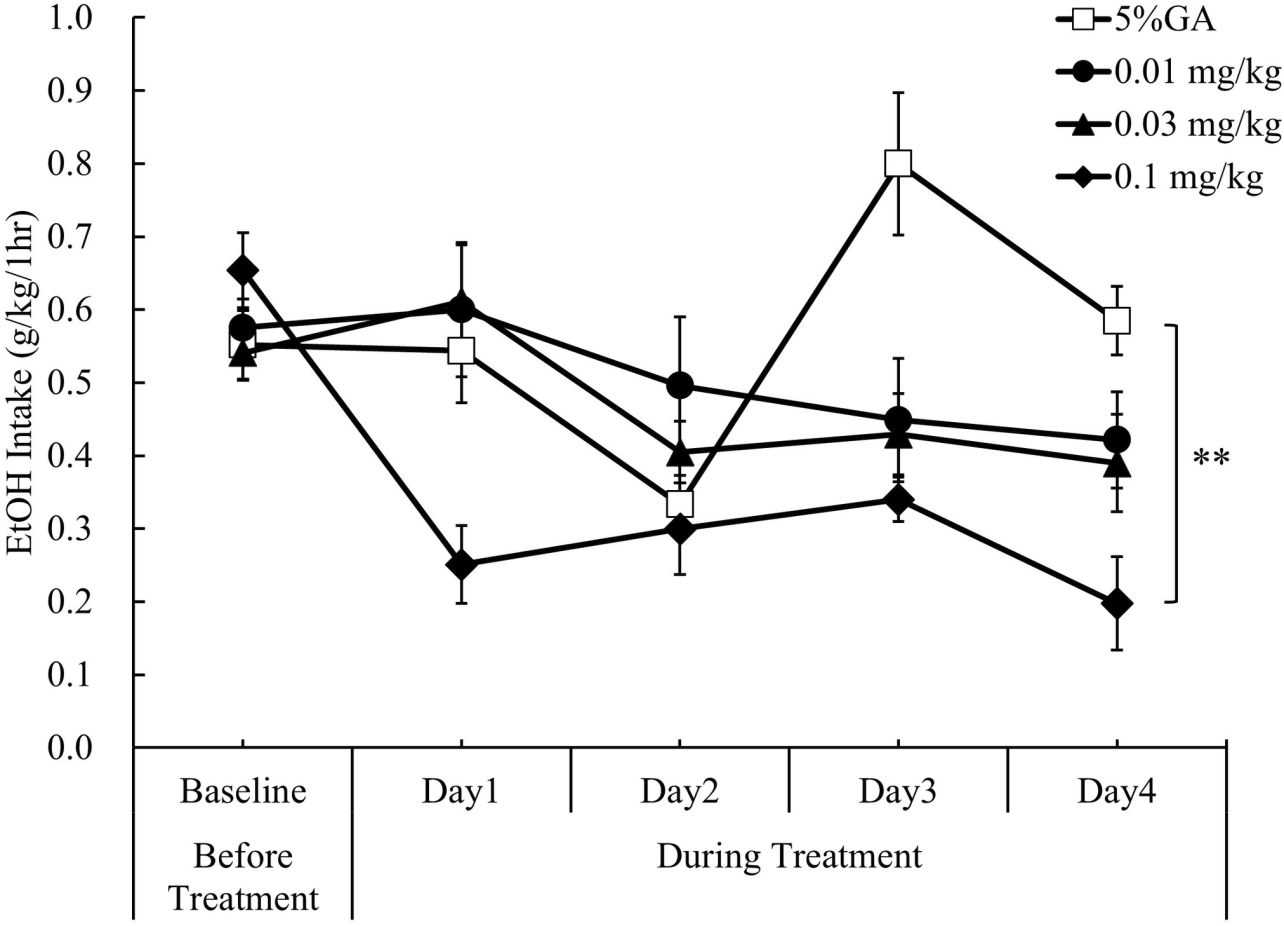
Effect of brexpiprazole on daily EtOH intake fluctuations in a limited access paradigm in rats. Differences in daily EtOH intakes over the 4‐day treatment period between 5% gum arabic and brexpiprazole (0.01, 0.03, or 0.1 mg/kg, p.o.) treatment were analyzed using a mixed effect model for repeated measures (MMRM), with animal ID, time (day), and treatment condition included as factors, and baseline EtOH intake (average over the 4 days before treatment) included as a covariate. Comparisons with 5% gum arabic were performed using Dunnett's multiple comparison procedure. The treatment of brexpiprazole at 0.1 mg/kg (p.o.) significantly decreased daily EtOH intakes compared to 5% gum arabic treatment over the consecutive 4‐day treatment period in limited access paradigm in rats. Data are presented as mean ± SEM (*n* = 5–6). **p < 0.01 vs. 5% gum arabic. 5% GA, 5% gum arabic solution.

With regard to effects on daily EtOH intakes, brexpiprazole at 0.1 mg/kg (p.o.) significantly decreased daily EtOH intakes over the 4‐day treatment period (*p* = 0.0022; Figure S2), while 0.01 and 0.03 mg/kg (p.o.) did not significantly decrease. Moreover, there was a significant difference in daily EtOH intake fluctuation for consecutive 4‐day treatment periods between 5% gum arabic treatment and brexpiprazole 0.1 mg/kg (p.o.) treatment (*p* = 0.0007; Figure 5).

Based on these results, 0.01 mg/kg (p.o.) was defined as a subeffective dose of brexpiprazole on EtOH intake in the limited access paradigm.

Each vehicle [saline (s.c.) and 5% gum arabic (p.o.)] did not influence the average EtOH intake for 4 days during treatment compared with before treatment, indicating that the decreasing effect of both compounds on EtOH intake would not be a nonspecific response associated with the administration procedure.

### 3.2 Combination Effect of Nalmefene With Brexpiprazole

Using the subeffective doses of nalmefene (0.04 mg/kg, s.c.) and brexpiprazole (0.01 mg/kg, p.o.), the combination effect of these compounds on daily EtOH intakes for 4 days was evaluated.

**FIGURE 6:**
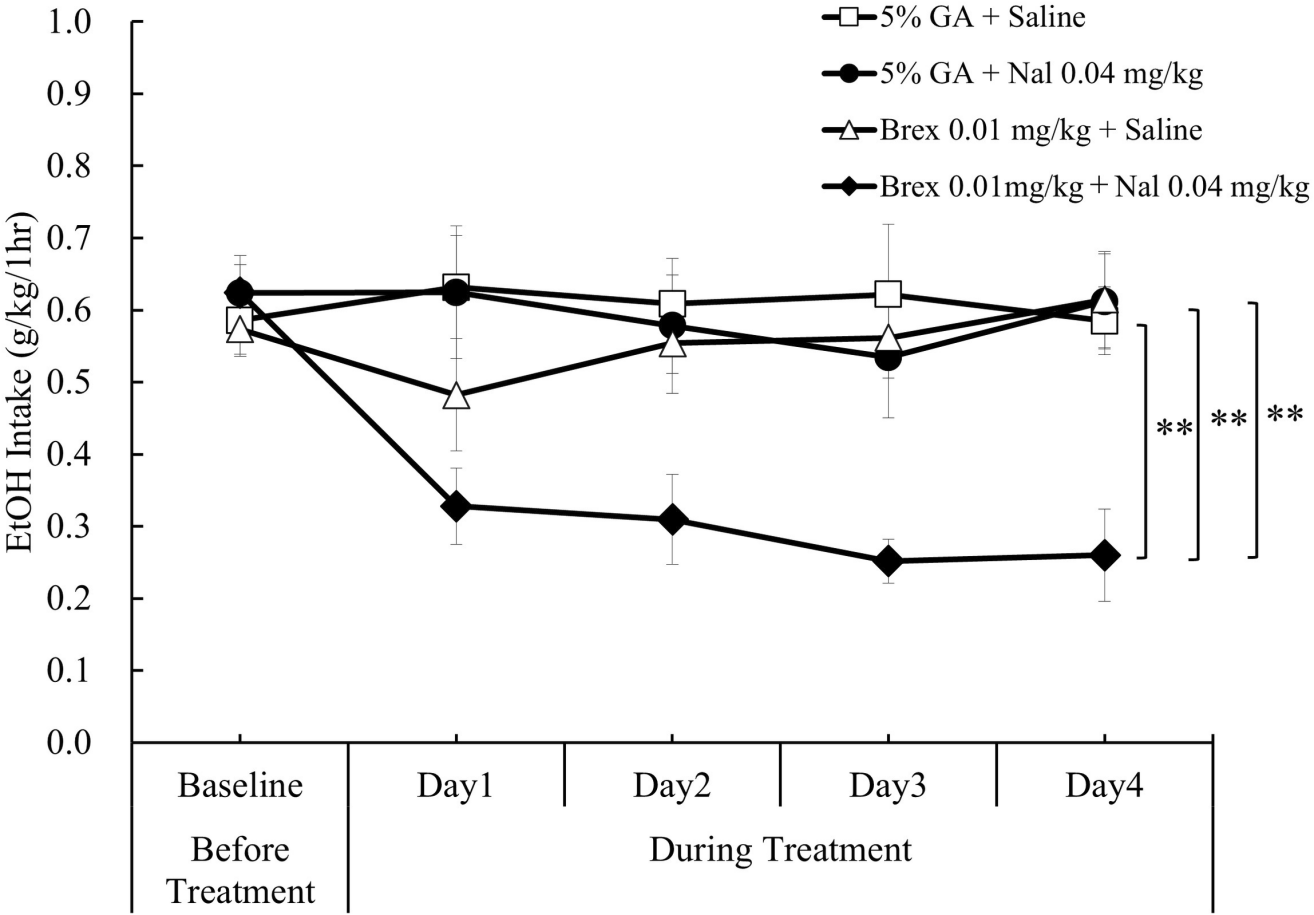
Combination effect of nalmefene with brexpiprazole on daily EtOH intakes in limited access paradigm in rats. Differences in daily EtOH intakes over a 4‐day treatment period between brexpiprazole (0.01 mg/kg, p.o.) + nalmefene (0.04 mg/kg, s.c.) combination treatment and other treatment conditions [5% gum arabic (p.o.) + saline (s.c.), 5% gum arabic (p.o.) + nalmefene (0.04 mg/kg, s.c.), and brexpiprazole (0.01 mg/kg, p.o.) + saline (s.c.)] were analyzed using a mixed effect model for repeated measures (MMRM), with animal ID, time (day), and treatment condition as factors, and baseline EtOH intake (average intake over the 4 days prior to treatment) as a covariate. Comparisons with the combination treatment were performed using Dunnett's multiple comparison procedure. The brexpiprazole (0.01 mg/kg, p.o.) + nalmefene (0.04 mg/kg, s.c.) combination treatment significantly decreased daily EtOH intakes compared to other treatment conditions over the consecutive 4‐day treatment period in limited access paradigm in rats. Data are presented as mean ± SEM (*n* = 6). **p < 0.01 vs. brexpiprazole 0.01 mg/kg (p.o.) + nalmefene 0.04 mg/kg (s.c.). 5% GA, 5% gum arabic solution; Brex, brexpiprazole; Nal, Nalmefene.

Only the combination treatment of brexpiprazole (0.01 mg/kg, p.o.) and nalmefene (0.04 mg/kg, s.c.) significantly decreased daily EtOH intakes (*p* = 0.001; Figure S3). There were significant differences in daily EtOH intake fluctuations for consecutive 4‐day treatment period between brexpiprazole (0.01 mg/kg, p.o.) + nalmefene (0.04 mg/kg, s.c.) combination treatment and other treatment conditions [5% gum arabic (p.o.) + saline (s.c.) combination treatment, 5% gum arabic (p.o.) + nalmefene (0.04 mg/kg, s.c.) combination treatment, and brexpiprazole (0.01 mg/kg, p.o.) + saline (s.c.) combination treatment] (each *p* < 0.0001; Figure 6).

Percent reductions of the average EtOH intake for the 4 days during the individual combination treatment from 4 days before the treatment were as follows: 5% gum arabic (p.o.) + saline (s.c.) combination treatment, −4.5%; 5% gum arabic (p.o.) + nalmefene (0.04 mg/kg, s.c.) combination treatment, 5.9%; brexpiprazole (0.01 mg/kg, p.o.) + saline (s.c.) combination treatment, 3.5%; and brexpiprazole (0.01 mg/kg, p.o.) + nalmefene (0.04 mg/kg, s.c.) combination treatment, 54.0%.

**FIGURE 7:**
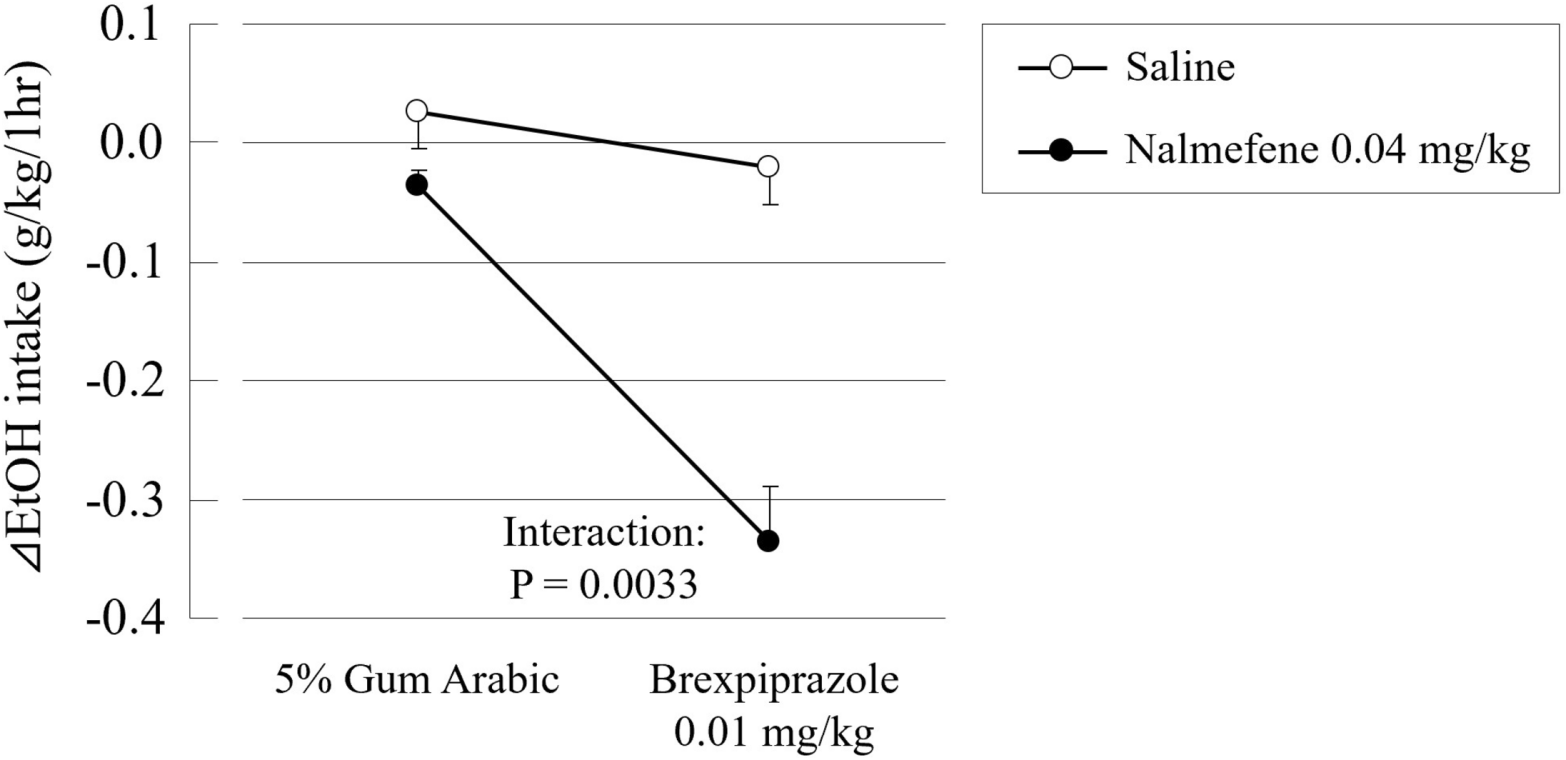
Combination effect of nalmefene with brexpiprazole on the delta values of the average EtOH intake in the limited access paradigm in rats. Data are presented as mean ± SEM (*n* = 6). Delta values were calculated for average EtOH intake over 4 days, comparing the pre‐treatment and treatment periods for each animal. Statistical analysis was performed using two‐way ANOVA with brexpiprazole (0.01 mg/kg, p.o.) and nalmefene (0.04 mg/kg, s.c.) (presence or absence) as factors. The combination of these compounds significantly and synergistically decreased the average EtOH intake for 4 days compared with before the treatment (interaction effect: *p* = 0.0033).

In addition, the delta values of the average EtOH intake for 4 days between before and during the treatment which were calculated in each animal. As shown in Figure 7, the combination of these compounds significantly and synergistically decreased the average EtOH intake for 4 days compared with before the treatment (interaction effect: *p* = 0.0033 by two‐way ANOVA).

### 3.3 Effects of Nalmefene and Brexpiprazole on Spontaneous Locomotor Activity

**FIGURE 8:**
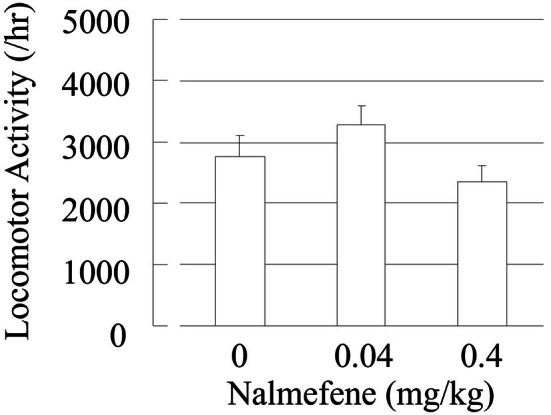
Effects of nalmefene on spontaneous locomotor activity in rats. Data are presented as mean ± SEM (*n* = 5–6). Nalmefene at doses of 0.04 and 0.4 mg/kg (s.c.) did not show any significant effect on spontaneous locomotor activity in rats compared with vehicle (saline) by Dunnett's test.

The effect of nalmefene at 0.04 and 0.4 mg/kg, s.c. on spontaneous locomotor activity was evaluated. As shown in Figure 8, nalmefene at both doses did not significantly decrease spontaneous locomotor activity. This result indicates that the effect of nalmefene on EtOH intake would not be caused by a sedating effect.

The effect of brexpiprazole on spontaneous locomotor activity has previously been reported, in which ED_50_ of brexpiprazole to suppress spontaneous locomotor activity was 3.4 mg/kg (p.o.) [18]. We also confirmed that brexpiprazole at 0.1 mg/kg (p.o.) did not decrease spontaneous locomotor activity in Wistar rats (data not shown). Therefore, it is considered that the effect of brexpiprazole on EtOH intake would not also be caused by a sedating effect.

## 4 Discussion

As previously reported [17], treatment of nalmefene alone decreased rat EtOH intake at 0.4 mg/kg (s.c.) in our study, which would be associated with the modulatory activity at opioid receptors. Nalmefene is an antagonist at the μ and the δ opioid receptors and a partial agonist at the κ opioid receptor [7]. It has been known that EtOH increases β‐endorphin and dynorphin releases in the brain [20]. β‐endorphin has affinity to the μ and the δ opioid receptors, while dynorphin has affinity to the κ opioid receptor. Mu and δ opioid receptor selective antagonists are known to reduce alcohol self‐administration in animals, whereas dynorphin and the κ opioid receptor system is supposed to be involved in negative reinforcing effects of alcohol [21]. The efficacy of nalmefene has been clinically confirmed in phase 3 trials and nalmefene is approved for the treatment of AUD patients [4, 7, 8, 22].

Treatment with brexpiprazole alone also decreased rat EtOH intake at 0.1 mg/kg (p.o.). This dose of brexpiprazole was much lower than its effective doses in other behavioral pharmacology studies already reported [13, 18]. Brexpiprazole acts as a partial agonist at the serotonin 5‐HT_1A_ receptor and the dopamine D_2_ receptor, and an antagonist at the 5‐HT_2A_ receptor and the adrenaline α_1B/2C_ receptors [13]. Brexpiprazole's activities at these receptors might be involved in its suppressive effects on EtOH intake.

It has been known that acute alcohol consumption increases dopamine release in the striatum, whereas chronic alcohol consumption may reduce dopamine receptor availability in the striatum [23]. Decrease of the D_2_ receptor availability and mRNA expression in the brain has been reported in AUD patients and in rats with long‐term alcohol consumption, respectively [23, 24]. There might be both cases of hypo‐dopaminergic and hyper‐dopaminergic conditions depending on the length of alcohol dependence in individual patients. These may indicate that modulation of dopamine signal activity by a D_2_ receptor partial agonist, which stimulates D_2_ receptors in the hypo‐dopaminergic conditions but also blocks D_2_ receptors in the hyper‐dopaminergic conditions, is more adequate for treatment of alcohol dependence than merely stimulating with a D_2_ receptor full agonist or blocking with a full antagonist.

It has been reported that chronic treatment of a serotonin 5‐HT_1A_ agonist or partial agonist suppresses EtOH intake in mice [25]. Increased α_1B_ adrenoceptor mRNA in the amygdala has been reported in patients with AUD [26]. In addition, co‐administration of an α_1B_ adrenoceptor antagonist and a serotonin 5‐HT_2A_ receptor antagonist inhibited EtOH intake and reversed EtOH preference in mice [27]. Brexpiprazole has almost similar affinity to the D_2_ receptor, the 5‐HT_1A_ receptor, the 5‐HT_2A_ receptor, and the α_1B_ adrenoceptor [13, 28]. Therefore, brexpiprazole's partial agonist and antagonist activities at these monoamine receptors may be simultaneously contributing to the suppression of EtOH intake.

Comorbidity of alcohol dependence is often reported in patients with psychiatric disorders such as schizophrenia, anxiety disorder, depressive disorder, and PTSD [9, 10, 11, 12]. Therefore, brexpiprazole's suppressive effect on EtOH intake may contribute to the treatment of patients with psychiatric disorders and improvement of their QOL. Moreover, the association of alcohol intoxication with cognitive impairment in dementia such as Alzheimer's disease (AD) has been considered, which was experimentally confirmed in transgenic AD mice repeatedly given alcohol [29]. Therefore, there might be benefits of treatment of alcohol dependence by brexpiprazole in people who have comorbidity of dementia, such as AD, and alcohol dependence.

Furthermore, the significant and synergistic combination effect of brexpiprazole and nalmefene was confirmed in our study. It seems that nalmefene does not show drug–drug interactions with other drugs [5]. Nalmefene is metabolized by UGT2B7, UGT1A3, 1A8, and CYP3A4/5 [5]. Brexpiprazole shows weak inhibitory activity on the CYP3A4 enzyme (IC_50_ > 13 μM, albeit data was from human enzymes) [30]. The plasma concentration (*C*_max_) of brexpiprazole at 1 mg/kg (p.o.) treatment in rats is 10 nM [31]. These suggest that brexpiprazole is unlikely to inhibit CYP3A4 at the dose (0.01 mg/kg, p.o.) tested in our study. In addition, main enzymes to metabolize brexpiprazole are CYP enzymes, suggesting that it is also unlikely to compete with the UGT enzyme metabolism of nalmefene. These indicate no potential drug–drug interactions between nalmefene and brexpiprazole in our study. Considering that mechanisms of action of these two compounds are different, combination effect of these compounds may make a new treatment option for AUD patients.

In conclusion, these results suggest that brexpiprazole, similar to nalmefene, may have the potential to reduce alcohol consumption in patients with alcohol dependence and may expand therapeutic potential for patients suffering from substance use disorders such as alcohol dependence by combining with nalmefene.

## Author Contributions

N.A. authored the paper. K.M. authored the paper, conceived and designed the study, performed experiments. M.N. performed experiments. Y.K. was involved in statistical analyses and was responsible for statistics. Y.O., M.S., and T.F. reviewed the paper. All authors contributed to the finalization of the paper and had final responsibility for the decision to submit for publication, took part in either drafting and/or revising the paper, and approved the final version of the paper.

## Funding

This work was supported by Otsuka Pharmaceutical.

## Disclosure

Registry and the Registration No. of the Study/Trial: The authors have nothing to report. Animal studies: The care and handling of the animals was in accordance with “Guidelines for Animal Care and Use in Otsuka Pharmaceutical Co Ltd.”

## Ethics Statement

The experimental procedure in this study was approved and conducted in accordance with Guidelines for Animal Care and Use in Otsuka Pharmaceutical Co Ltd.

## Consent

The authors have nothing to report.

## Conflicts of Interest

All of the authors are employees and own stock of Otsuka Holdings Co. Ltd.

## Supporting information


**Data S1:** npr270107‐sup‐0001‐DataS1.zip.

## Data Availability

The data that supports the findings of this study are available in the Supporting Information of this article.
