# Supplementary material for: Synergistic Effect of Combination Treatment of Brexpiprazole and Nalmefene on Ethanol Intake in Rats
Source: Neuropsychopharmacol Rep. 2026 Mar 6;46(1):e70107. doi: 10.1002/npr2.70107 (PMC12966623; doi:10.1002/npr2.70107)
Supplement: Supplementary file 1 — Data S1: npr270107‐sup‐0001‐DataS1.zip. [file NPR2-46-e70107-s001.zip › npr270107-sup-0002-Supinfo1.docx]

**
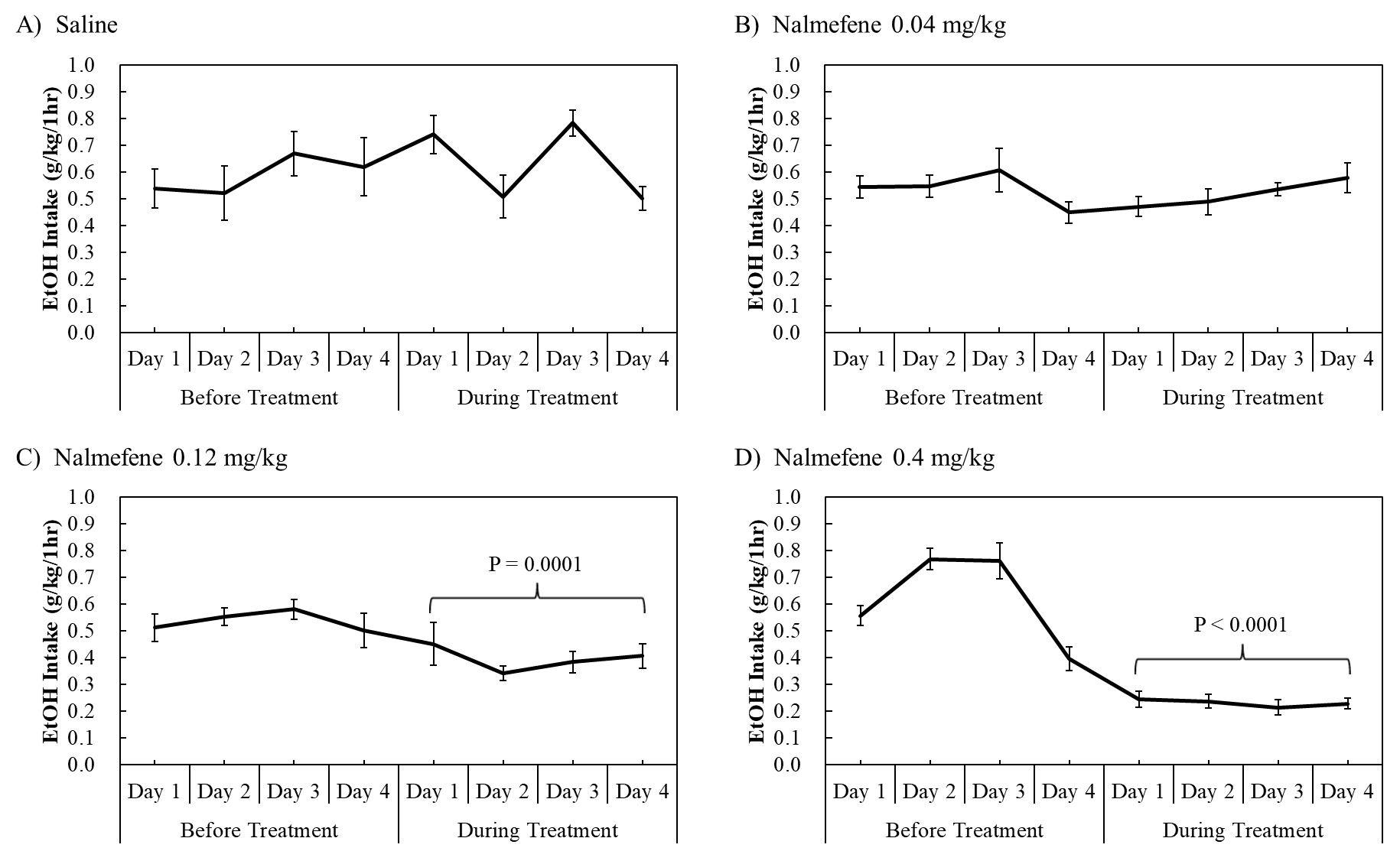
**

**Supplemental Figure 1. Effect of nalmefene on daily EtOH intakes in limited access paradigm in rats**

EtOH intakes in (A) saline treatment, (B) nalmefene 0.04 mg/kg treatment, (C) nalmefene 0.12 mg/kg treatment, and (D) nalmefene 0.4 mg/kg treatment. Data are presented as mean ± SEM (n = 5 - 6). The differences of daily EtOH intakes between consecutive 4 days before and 4 days during the treatment of saline (s.c.) or nalmefene (0.04, 0.12, 0.4 mg/kg, s.c.) were analyzed using a mixed effect model for repeated measures (MMRM), with animal ID, time (day), and treatment period (pre-treatment or treatment) included as factors. Nalmefene at doses of 0.12 and 0.4 mg/kg (s.c.) indicated the statistically significant decreasing effect on daily EtOH intakes in limited access paradigm in rats (p = 0.001 and p < 0.0001, respectively).


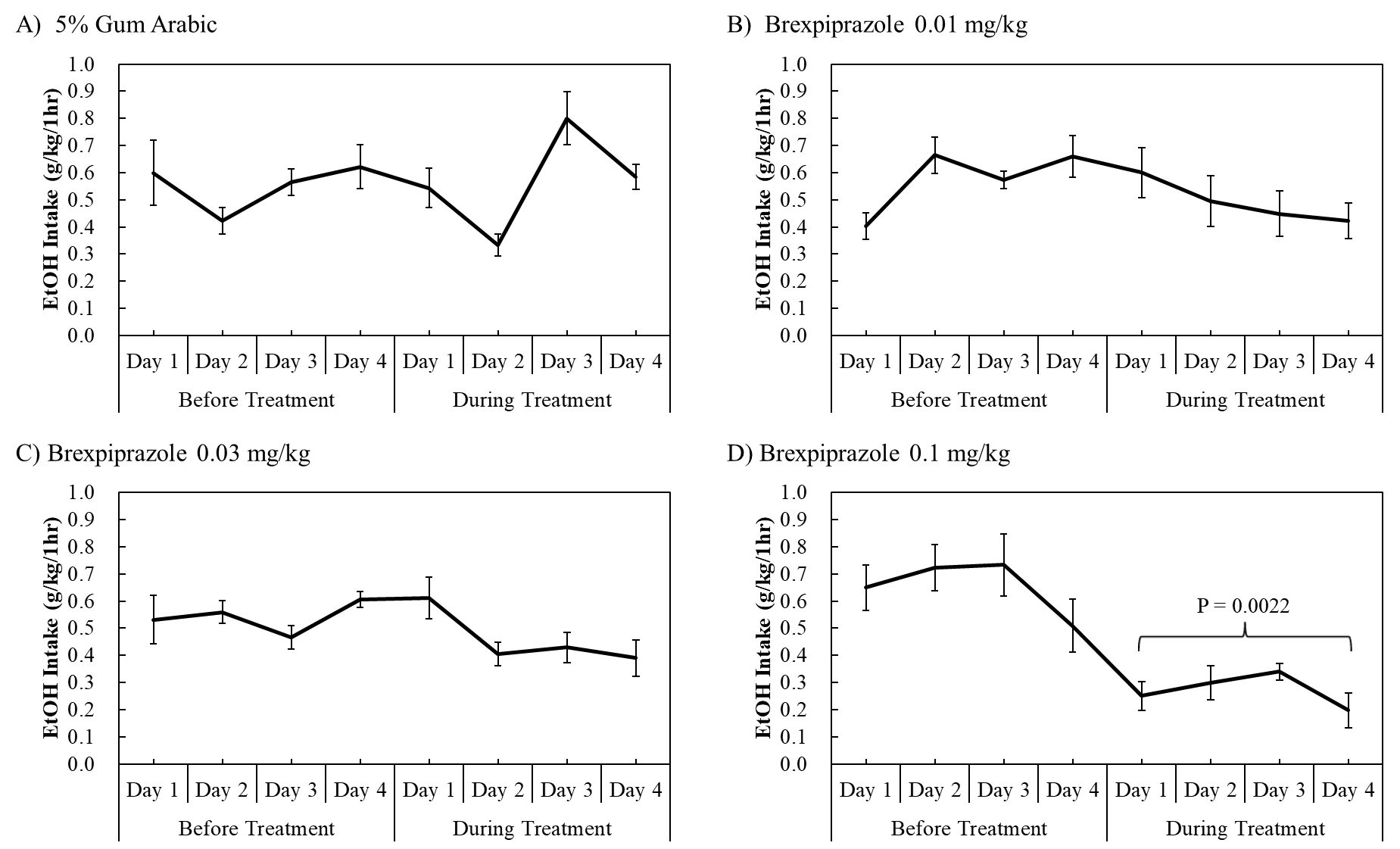


**Supplemental Figure 2. Effect of brexpiprazole on daily EtOH intakes in limited access paradigm in rats**

EtOH intakes in (A) 5% gum arabic treatment, (B) brexpiprazole 0.01 mg/kg treatment, (C) brexpiprazole 0.03 mg/kg treatment, and (D) brexpiprazole 0.1 mg/kg treatment. Data are presented as mean ± SEM (n = 5 - 6). The differences of daily EtOH intakes between consecutive 4 days before and 4 days during the treatment of 5% gum arabic (p.o.) or brexpiprazole (0.01, 0.03, 0.1 mg/kg, p.o.) were analyzed using a MMRM, with animal ID, time (day), and treatment period (pre-treatment or treatment) included as factors. Brexpiprazole at doses of 0.1 mg/kg (p.o.) indicated the statistically significant decreasing effect on daily EtOH intakes in limited access paradigm in rats (p = 0.0022).


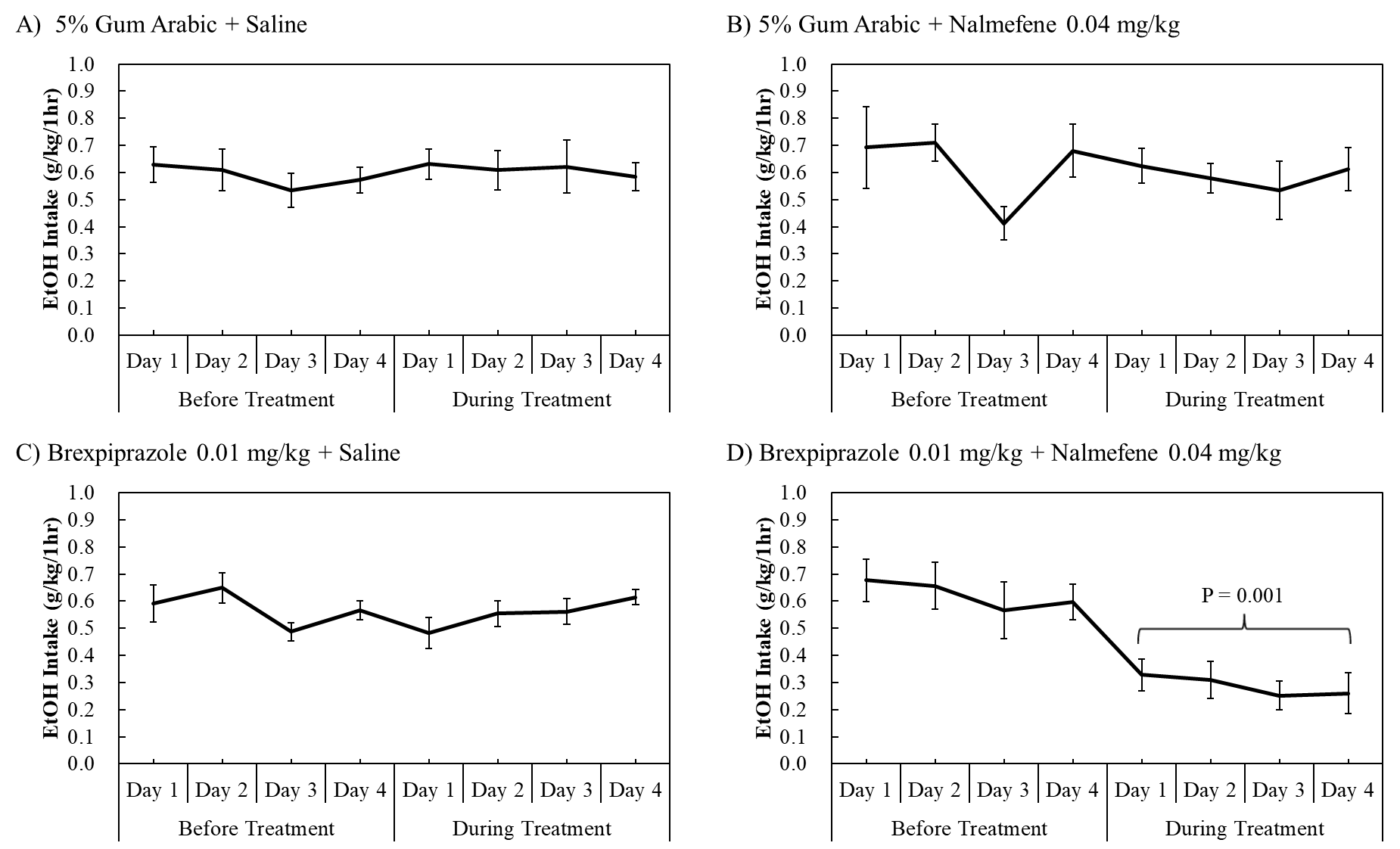


**Supplemental Figure 3. Combination effect of nalmefene and brexpiprazole on daily EtOH intakes in limited access paradigm in rats**

EtOH intakes in (A) 5% gum arabic (p.o.) + saline (s.c.) combination treatment, (B) 5% gum arabic (p.o.) + nalmefene (0.04 mg/kg, s.c.) combination treatment, (C) brexpiprazole (0.01 mg/kg, p.o.) + saline (s.c.) combination treatment, and (D) brexpiprazole (0.01 mg/kg, p.o.) + nalmefene (0.04 mg/kg, s.c.) combination treatment. Data are presented as mean ± SEM (n = 6). The differences of daily EtOH intakes between consecutive 4 days before and 4 days during each combination treatment were analyzed using a MMRM, with animal ID, time (day), and treatment period (pre-treatment or treatment) included as factors. Only the combination treatment of brexpiprazole (0.01 mg/kg, p.o.) and nalmefene (0.04 mg/kg, s.c.) significantly decreased daily EtOH intakes (p = 0.001).
